# PTPN21 inhibits cell apoptosis of acute lymphoblastic leukemia induced by chemotherapeutic agents via GADD45A and JNK signaling pathway

**DOI:** 10.1371/journal.pone.0322273

**Published:** 2025-04-30

**Authors:** Chenze Zhao, Yu Zhang, Linlin Huang, Jingjing Xiang, Baodong Ye, Ni Zhu

**Affiliations:** 1 Heart Center, Department of Cardiovascular Medicine, Zhejiang Provincial People’s Hospital, Affiliated People’s Hospital, Hangzhou Medical College, Hangzhou, Zhejiang, China; 2 Department of Hematology, The First Affiliated Hospital of Zhejiang Chinese Medical University (Zhejiang Provincial Hospital of Chinese Medicine), Hangzhou, Zhejiang, China; Rutgers: Rutgers The State University of New Jersey, UNITED STATES OF AMERICA

## Abstract

Acute lymphoblastic leukemia (ALL), a hematologic malignancy characterized by the uncontrolled proliferation of immature lymphocytes, often results in unfavorable long-term survival prospects for patients. PTPN21, a protein with established roles in oncogenesis, has been implicated in the pathogenesis of ALL. This study explores the role of PTPN21 in ALL cell apoptosis induced by chemotherapeutic agents. Key findings reveal that elevated PTPN21 levels hinder the apoptosis of ALL cells in response to vincristine (VCR) and daunorubicin (DNR). PTPN21 accomplishes this by inhibiting the GADD45A and JNK signaling pathways, thereby reversing cell cycle arrest in the G2/M phase. Restoring GADD45A reverses the anti-apoptotic effect of PTPN21. These findings suggest that targeting PTPN21 in conjunction with chemotherapy may represent a novel therapeutic strategy for ALL, offering potential implications for improving treatment efficacy and overcoming drug resistance.

## Introduction

Acute lymphoblastic leukemia (ALL) is a hematologic malignancy characterized by the abnormal proliferation of immature lymphocytes in the bone marrow, blood, and other organs [1]. In recent years, the optimization of chemotherapy doses and regimens has increased the cure rate to 90% in pediatric patients with ALL [2]. Although progress has been made in treating adult ALL, some patients (approximately 10%) do not respond to initial chemotherapy, and more than half eventually relapse [3]. The remission rate for adult patients with ALL is only 20–40% [4].

Vincristine (VCR) and daunorubicin (DNR) are pivotal in the treatment of ALL [5,6]. However, VCR is frequently associated with the onset of peripheral neuropathy, a significant adverse effect [5]. Investigating the mechanism through which VCR acts in ALL treatment, identifying target genes that influence the therapeutic efficacy of VCR, and enhancing the sensitivity of ALL to VCR are crucial steps toward mitigating its neurotoxic side effects.

PTPN21 encodes protein tyrosine phosphatase nonreceptor 21, which was initially discovered in human skeletal muscle [9]. Recent reports have highlighted the altered expression of PTPN21 in the development of various cancers, such as bladder cancer [10], gastric cancer [11], pancreatic carcinoma [12], and glioma [13]. In recent years, several studies have emphasized the role of abnormal expression and mutation of protein tyrosine kinases (PTKs) and protein tyrosine phosphatases (PTPs) in leukemia [7,8]. Specifically, several studies have investigated the role of PTPN21 in ALL. Xiao H et al. [14] conducted a comprehensive whole-exome sequencing analysis in three adult patients with Ph- B-cell ALL (Ph- B-ALL) using samples from diagnosis to relapse after allogeneic hematopoietic stem cell transplantation (allo-HSCT) and discovered PTPN21 mutations in the samples obtained during relapse. Wang H et al. [15] revealed that the endogenous PTPN21-CDSlong isoform hindered the destruction of ALL cells by NK cells through regulation of the KIR-HLA-I axis. Nevertheless, it remains unclear whether PTPN21 impacts the effects of VCR and DNR on acute lymphoblastic leukemia.

P53 is a crucial tumor suppressor protein, and its role in leukemia is primarily manifested in regulating cell apoptosis and autophagy. Studies have demonstrated that the P53 signaling pathway plays a pivotal role in both acute myeloid leukemia (AML) and chronic lymphocytic leukemia (CLL). For instance, mutations or functional defects in P53 are closely associated with disease progression and prognosis in CLL [16]. Additionally, P53 induces cell apoptosis by activating downstream genes such as GADD45A [17]. The JNK (c-Jun N-terminal kinase) pathway is significant in cellular stress responses and apoptosis. Research has found that the JNK pathway promotes cell apoptosis by phosphorylating and activating downstream effectors [18]. For example, in certain leukemia cells, the activation of the JNK pathway is closely related to cell apoptosis [19].

GADD45A (growth arrest and DNA damage-inducible 45α) is a stress-responsive gene that plays a critical role in DNA damage and cell cycle arrest. Studies indicate that GADD45A can be activated through the P53 signaling pathway and further promote cell apoptosis [20]. Research has found that GADD45A expression is upregulated in samples from patients with chronic myeloid leukemia (CML) in the chronic phase, but downregulated in the accelerated and blast phases, which correlates with the severity of the disease, and low expression predicts a poorer prognosis. Furthermore, GADD45A inhibits cell proliferation and promotes apoptosis by regulating AKT, p38, and Stat5 signaling pathways, thereby exerting its tumor suppressive effects [21]. In AML, a decrease in the methylation level of the GADD45A promoter is associated with poor prognosis [22]. Studies have also revealed that the absence of GADD45A accelerates the development of BCR-ABL-driven CML, leading to more severe disease and higher numbers of leukemic stem cells. Additionally, high expression of GADD45A in AML is associated with shorter progression-free survival, suggesting that it may promote the survival of malignant hematopoietic cells by maintaining genomic stability [23].

Vincristine exerts inhibitory effects on cancer cells by inhibiting microtubule assembly and blocking cell cycle progression. Its primary mechanisms include disrupting the formation of mitotic spindle microtubules, resulting in abnormal cell division and proliferation. In ALL, vincristine reduces the number of leukemic cells by inhibiting their mitosis, thereby achieving therapeutic effects [24,25]. Studies have shown that vincristine may induce drug resistance in certain ALL cell lines. For example, ICN13 BCP-ALL cells develop resistance to vincristine in the presence of supportive microenvironments, with significant changes in the expression of specific proteins identified through proteomic analysis [26].

Daunorubicin, an anthracycline drug, inhibits the growth and division of cancer cells by inserting into DNA double strands and interfering with DNA replication and transcription [27]. However, the use of daunorubicin is often accompanied by severe bone marrow suppression and infection risks. For instance, liposomal daunorubicin exhibits lower non-hematological toxicity but may be associated with a higher infection rate [28]. Additionally, glucocorticoid-resistant pediatric ALL samples exhibit alterations in splicing patterns (such as FLT3 mutations), which may affect drug efficacy [29].

This study revealed that elevated PTPN21 expression impairs apoptosis in ALL cells in response to chemotherapeutic drugs, such as VCR and DNR. Through a series of in vitro experiments, we sought to elucidate the underlying mechanisms by which VCR acts during the treatment of ALL. Additionally, our research supports targeting PTPN21 as a therapeutic strategy for ALL management.

## Materials and methods

### Cell culture

The human acute lymphoblastic leukemia cell lines Jurkat and NALM-6 were acquired from the Cell Bank of the Shanghai Institute of Biochemistry and Cell Biology, Chinese Academy of Sciences (Shanghai, China). These cells were cultured in Roswell Park Memorial Institute 1640 medium (Gibco, Carlsbad, CA, USA) supplemented with 10% fetal bovine serum (Gibco, Carlsbad, CA, USA). HEK293T cells were cultured in Dulbecco’s Modified Eagle’s Medium supplemented with 10% fetal bovine serum and 4.5 g/L glucose (Gibco, Carlsbad, CA, USA). All cells were maintained at 37 °C in a humidified atmosphere with 5% CO2.

### Chemicals and antibodies

Vincristine (VCR) and daunorubicin (DNR) were purchased from Selleck Chemicals (Houston, TX, USA). The following primary antibodies were used: rabbit monoclonal DYKDDDDK tag (FLAG) and PTPN21 antibodies were obtained from Abcam (Boston, MA, USA); rabbit monoclonal Bax and mouse monoclonal Bcl-2 antibodies were obtained from Thermo Fisher Scientific (Waltham, MA, USA); rabbit polyclonal cleaved caspase 3 antibody was obtained from Cell Signaling Technology (Beverly, MA, USA); rabbit monoclonal P53 and rabbit polyclonal Jun antibodies were obtained from Proteintech Group (Wuhan, China). Mouse monoclonal p-P53 (Ser15) antibody, rabbit polyclonal JNK antibody, and p-SAPK/JNK (Thr183/Tyr185) antibody were purchased from Cell Signaling Technology (Beverly, MA, USA); rabbit monoclonal p-Jun (Ser63) antibody was obtained from Abcam (Boston, MA, USA); mouse polyclonal GAPDH antibody was obtained from Goodhere Biotech (Hangzhou, China). Horseradish peroxidase-labeled secondary antibodies, including goat anti-mouse and goat anti-rabbit, were obtained from Boster Biological Technology (Wuhan, China).

### Plasmids and transfection

The pHIV7/SFFV-GFP vector plasmid (S1 Fig) was a gift from Professor Jiing-Kuan Yee from the City of Hope National Medical Center (Duarte, CA, USA). By analyzing the FLAG Tag sequence, a single-stranded oligo containing FLAG Tag was designed and synthesized. The synthesized oligo was spliced into a complete gene sequence using PCR. We loaded the synthesized sequence into the pMD18-T vector, transformed it into DH5α cells, and used sequencing to verify whether the inserted sequence was correct in the recombinant clone. Next, the SFFV promoter was sequentially spliced with the PTPN21 gene sequence and FLAG Tag, and the pHIV7/SFFV-GFP plasmid was digested using BamHI and Bgl II enzymes. The fragments were recovered, and the spliced sequences were ligated to obtain the pHIV7/SFFV-PTPN21-3×FLAG plasmid (Supplementary material). The plasmid was transfected into HEK293T cells along with the lentiviral packaging plasmids pMD2. G and psPAX2 (Thermo Fisher Scientific, Waltham, MA, USA). After 48 hours, the viral suspension was collected and filtered through a 0.45 μm filter. The plasmid overexpressing GADD45A was purchased from Pinggu Biotechnology Co. (Wuhan, China). The plasmid overexpressing GADD45A was co-transfected with lentiviral packaging plasmids pCMV-dR8.9 and pCMV-VSV-G (Addgene, Cambridge, MA, USA) in 293T cells. The supernatant was collected after 48 hours and filtered through a 0.22 μm filter. The lentivirus in the supernatant was subsequently concentrated and purified, and the viral titer was determined. The target cells were transfected with a virus titer corresponding to a multiplicity of infection = 50. Transfection was verified using an anti-FLAG antibody and Western blot analysis.

### Apoptosis assay

Cells were pretreated with 5 μM vincristine (VCR), 100 nM daunorubicin (DNR) or DMSO (as a control) for 48 hours. Subsequently, the cells were collected and washed twice with cold PBS. Apoptosis was assessed using an apoptosis detection kit (KeyGEN Biotech, Nanjing, China). The cells were suspended in 100 × Annexin V binding buffer and incubated with Annexin V-PE or Annexin V-APC antibody and 7-aminoactinomycin D at room temperature for 15 minutes. Cell analysis was performed using a Beckman Coulter CytoFLex flow cytometer. Annexin V-positive cells were identified as apoptotic cells. Examples of gating strategies, single-color controls and negative control for the flow cytometry experiments are provided in the supplementary material and S2 Fig.

### Cell cycle assay

Cells were pretreated with 5 μM VCR, 100 nM DNR or DMSO (as a control) for 48 hours and then harvested for cell cycle analysis. The cells were fixed at 4 °C in precooled 70% ethanol for more than 4 hours. After two washesin precooled PBS, the cells were incubated with 50 μg/ml RNase at 37 °C for 30 minutes and were subsequently stained with 50 μg/ml PI (propidium iodide, Merck, Germany) at 4 °C for 30 minutes in the dark. The stained cells were resuspended in 500 μl of PBS for flow cytometry (FCM) detection.

### Reverse transcription-polymerase chain reaction (RT-qPCR)

Total RNA was extracted from cell samples using TRIzol solution (Thermo Fisher Scientific, Waltham, MA, USA), and cDNA was synthesized using HiScript II Q RT SuperMix for qPCR (Vazyme, R222-01, Nanjing, China) according to the manufacturer’s instructions. PCR was performed using ChamQ SYBR Color qPCR Master Mix (Low ROX Premixed) (Vazyme, Q431-02, Nanjing, China) and an ABI QuantStudio 6 Flex fluorogenic quantitative PCR instrument. The level of the target gene was normalized to that of the housekeeping gene GAPDH. The primer sets used were as follows: Homo GAPDH, 5`-TCAAGAAGGTGGTGAAGCAGG-3` (forward), 5`-TCAAAGGTGGAGGAGTGGGT-3` (reverse); Homo GADD45A, 5`-CTGGAGAGCAGAAGACCGAA-3` (forward), 5`-CAGCGTCGGTCTCCAAGA-3` (reverse); Homo ATF4, 5`-GGAAACCATGCCAGATGACC-3` (forward), 5`-GATCTGGAGTGGAGGACAGG-3` (reverse); and Homo PARP1, 5`-CCGCATACTCCATCCTCAGT-3` (forward), 5`-GCTATCATCAGACCCTCCCC-3` (reverse).

### Western blot analysis

Cells were harvested and subjected to lysis in radioimmunoprecipitation assay lysis buffer (Beyotime Institute of Biotechnology, Shanghai, China) containing a protein enzyme inhibitor cocktail (Beyotime Institute of Biotechnology, Shanghai, China) and a phosphatase inhibitor (Beyotime Institute of Biotechnology, Shanghai, China). This lysis process was performed on ice for 1 hour. The cell lysates were then centrifuged at 12,000 × g for 10 minutes at 4 °C. The supernatant was heated to 100 °C for 10 minutes after the addition of loading buffer (KeyGEN BioTech, Nanjing, China). Subsequently, the cell lysates were centrifuged once again at 12,000 × g for 10 minutes at 4 °C. Equal amounts of the prepared protein samples were loaded onto an SDS-PAGE (sodium dodecyl sulfate-polyacrylamide) gel, electrophoretically separated (110 V for 100 minutes), and transferred onto an Immobilon-P polyvinylidene difluoride membrane (Merck, Germany). The membranes were blocked with 5% nonfat milk (BD Pharmingen, San Diego, CA, USA) at room temperature for 2 hours. The membranes were then incubated with primary antibodies at 4 °C overnight, followed by incubation with secondary antibodies at room temperature for 1 hour. Finally, the protein bands were visualized using X-ray film and were developed with a developer and fixer.

### Coimmunoprecipitation (Co-IP)

The cells were harvested and lysed using NP-40 buffer (Beyotime Institute of Biotechnology, Shanghai, China) supplemented with 1 mmol/L phenylmethylsulfonyl fluoride (PMSF) (Servicebio Technology, Wuhan, China) and a protease inhibitor cocktail (Servicebio Technology, Wuhan, China). The cell lysates were then incubated with antibodies specific for Cdk1 (Proteintech Group, Wuhan, China), Cyclin B1 (Proteintech Group, Wuhan, China), PTPN21 (Abcam, Cambridge, UK), GADD45A (Proteintech, Wuhan, China), or IgG (Vazyme, Nanjing, China) as a control overnight at 4 °C. Next, the lysates were incubated with protein A/G beads (Thermo Fisher Scientific, Waltham, MA, USA) for six hours at the same temperature. The beads were subsequently washed 5 times with immunoprecipitation buffer. The immunoprecipitates were then separated by SDS-PAGE, transferred to membranes, and analyzed via western blot to detect the interaction between Cdk1 and Cyclin B1 or PTPN21 and GADD45A.

### RNA sequencing

Jurkat cells in both the control and PTPN21 overexpression groups were treated with vincristine (VCR) for 48 hours. Subsequently, cells from both groups were harvested for RNA sequencing (RNA-seq) with three replicates per group. RNA was extracted using TRIzol reagent (Thermo Fisher Scientific, Waltham, MA, USA). The digital gene expression tag profiling and data analysis were performed by an external commercial company (Pinggu Biotechnology Co., Wuhan, China). Based on the results of RNA sequencing, low-expression apoptosis-related genes were screened by the KEGG database gene set (pathway ID: hsa04210). The RNA-seq data used in our study have been deposited in the NCBI Gene Expression Omnibus and are accessible through GEO Series accession number GSE253979 (https://www.ncbi.nlm.nih.gov/geo/query/acc.cgi?acc=GSE253979).

### Statistical analysis

All independent experiments were replicated three times. Statistical analysis was performed using SPSS software, version 22.0 (IBM SPSS, NY, USA). Differences between mean values were assessed using one-way analysis of variance (ANOVA) and Tukey’s multiple comparisons test. The data are presented as the mean ± standard deviation, and a p value <0.05 indicated statistical significance.

## Results

### PTPN21 overexpression in ALL cell lines inhibited DNR- or VCR-induced cell apoptosis

**Fig 1:**
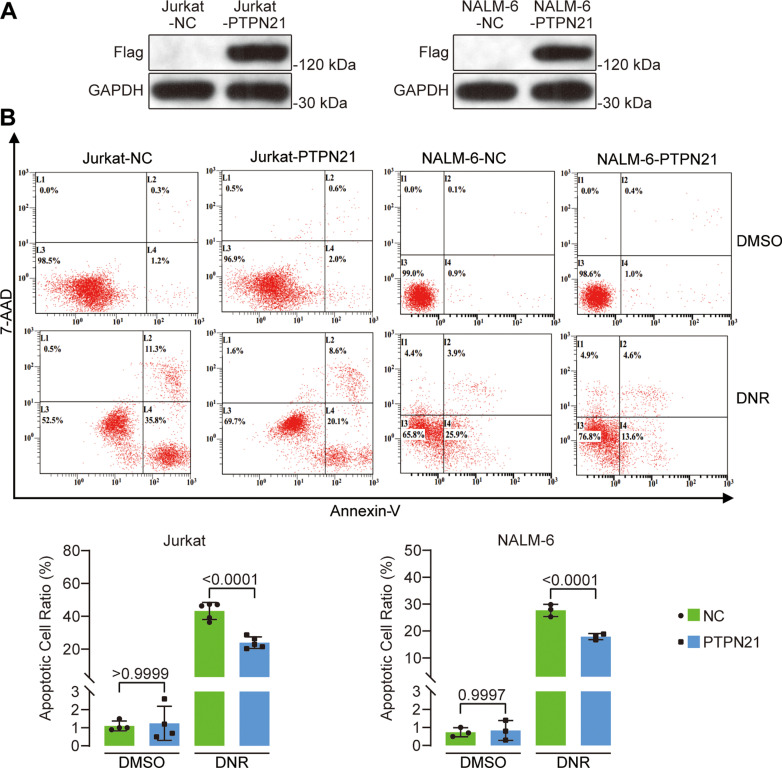
The impact of PTPN21 overexpression on ALL cell lines and apoptosis induced by DNR or VCR. **(A)** Western blot analysis confirmed PTPN21 overexpression using an anti-FLAG antibody. **(B)** Flow cytometry analysis of apoptosis in Jurkat (n = 5) and NALM-6 (n = 3) cells after 48 h of treatment with 100 nM DNR. The data are presented as the mean ± standard deviation, and a p value <0.05 indicated statistical significance.

**Fig 2:**
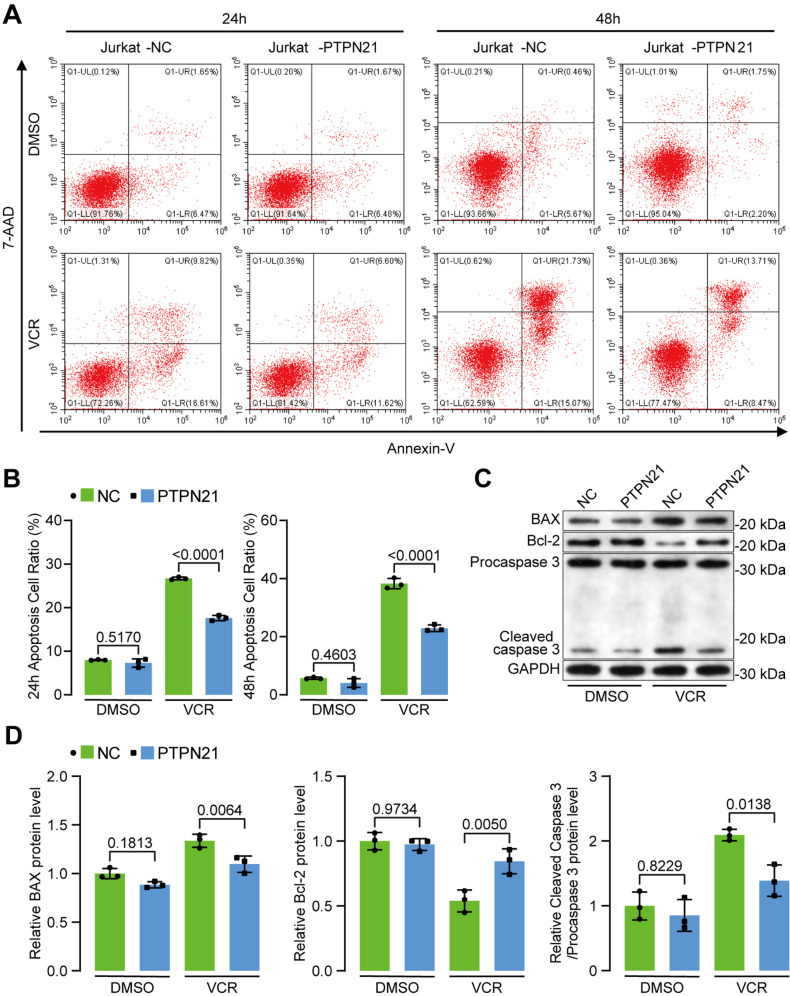
The impact of PTPN21 overexpression on Jurkat cell apoptosis induced by VCR. **(A)** Flow cytometry analysis of apoptosis in Jurkat cells after treatment with 5 μM VCR for 24 h or 48 h. **(B)** Apoptosis analysis for **(A)** (n = 3). **(C)** Western blot results showing the expression of apoptosis-related proteins, including Bax, Bcl-2, and caspase 3. **(D)** Protein data analysis results for **(C)** (n = 3). The data are presented as the mean ± standard deviation, and a p value <0.05 indicated statistical significance.

To investigate the role of PTPN21 in acute lymphoblastic leukemia (ALL), we overexpressed PTPN21 in Jurkat and NALM-6 cells, while cells transfected with the pHIV7-SFFV-GFP vector served as a control. Western blot with an anti-FLAG antibody confirmed the successful overexpression of PTPN21 (Fig 1A). We assessed apoptosis in the PTPN21 overexpression group and the control group using an Annexin V/7-AAD or APC/7-AAD double binding assay. No significant difference was observed in the proportion of apoptotic cells between the two groups. PTPN21 overexpression in Jurkat and NALM-6 cells had no impact on cell apoptosis. However, the PTPN21 overexpression group exhibited a significantly lower proportion of apoptotic cells induced by daunorubicin (DNR) (Jurkat: PTPN21 overexpression group vs. control group 23.94 ± 3.48 vs. 43.26 ± 5.14, *p* < 0.0001; NALM-6: PTPN21 overexpression group vs. control group 17.93 ± 1.12 vs. 27.70 ± 2.26, *p* < 0.0001; Fig 1B). In Jurkat cells, the proportion of apoptotic cells induced by vincristine (VCR) was significantly lower in the PTPN21 overexpression group than in the control group after 24 h or 48 h of treatment (24 h: PTPN21 overexpression group vs. control group 17.61 ± 0.60 vs. 26.70 ± 0.32, *p* < 0.0001; 48 h: PTPN21 overexpression group vs. control group 22.94 ± 1.15 vs. 38.29 ± 1.77, *p* < 0.0001; Fig 2A-B). After 48 h of treatment with VCR, PTPN21 overexpression inhibited the expression of Bax and cleaved caspase 3, while the expression of the antiapoptotic protein Bcl-2 was increased (Fig 2C-D). These results suggest that PTPN21 overexpression in ALL cell lines inhibits apoptosis induced by chemotherapeutic agents.

### PTPN21 overexpression inhibited the GADD45A and JNK signaling pathways

**Fig 3:**
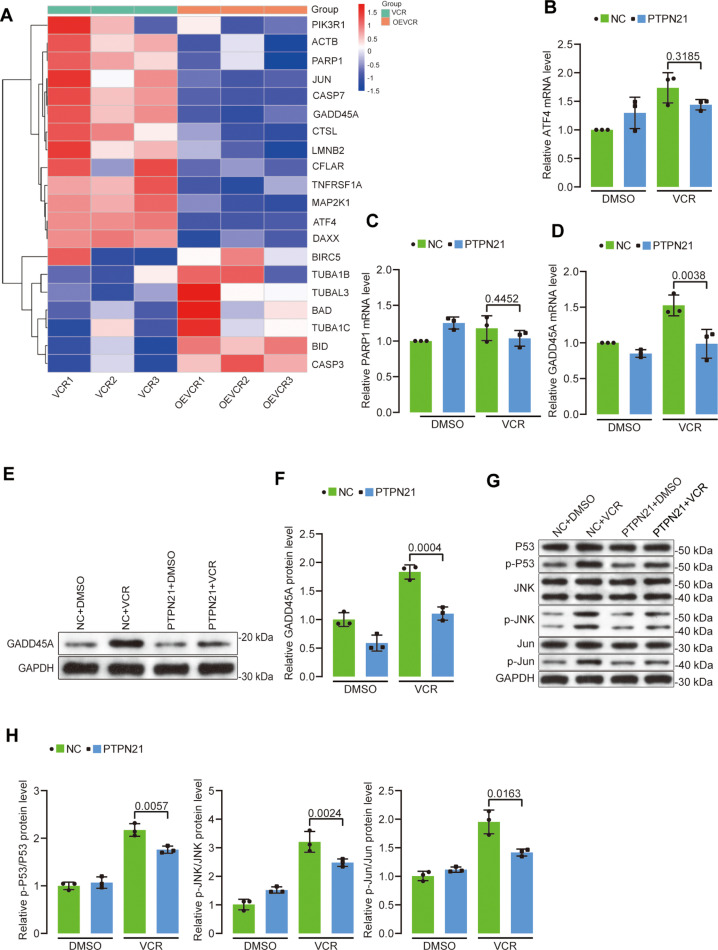
The influence of PTPN21 overexpression on GADD45A and the JNK signaling pathway. **(A)** Analysis of the effect of PTPN21 overexpression on the VCR-induced apoptosis pathway in Jurkat cells based on transcriptome sequencing results. “OEVCR” stands for the PTPN21 overexpression group. **(B-D)** Detection of the mRNA expression levels of GADD45A, ATF4, and PARP1 by RT-qPCR (n = 3). **(E)** Western blot analysis of GADD45A protein expression. **(F)** Protein data analysis results for **(E)** (n = 3). **(G)** Western blot results illustrating the phosphorylation status of P53 and proteins in the JNK signaling pathway. **(H)** Protein data analysis results for **(G)** (n = 3). The data are presented as the mean ± standard deviation, and a p value <0.05 indicated statistical significance.

To explore the pivotal molecules involved in the PTPN21-mediated inhibition of apoptosis, we conducted transcriptome sequencing of Jurkat cells after 48 h of treatment with VCR, while cells transfected with the pHIV7-SFFV-GFP vector served as a control. Analysis of the apoptotic pathway revealed a decrease in the expression of a series of apoptosis-related genes, including PIK3R1, ACTB, PARP1, JUN, CASP7, GADD45A, CTSL, LMNB2, CFLAR, TNFRSF1A, MAP2K1, ATF4, and DAXX, in the PTPN21 overexpression group (Fig 3A). A literature review established that GADD45A [20], ATF4 [30], and PARP1 [31] are promoters of apoptosis. Particularly, GADD45A, a stress-responsive gene critical in DNA damage and cell cycle arrest, is activated by the P53 pathway to promote cell apoptosis. Consequently, we investigated the expression of these genes in Jurkat cells. RT-qPCR analysis revealed that PTPN21 overexpression decreased the mRNA levels of GADD45A, ATF4, and PARP1 in VCR-treated Jurkat cells. However, only the changes in GADD45A were statistically significant (PTPN21 overexpression group vs. control group 0.988 ± 0.202 vs. 1.525 ± 0.147, *p* < 0.01; Fig 3B-D). Western blot results further confirmed that PTPN21 significantly reduced the increased protein levels of GADD45A induced by VCR (NC + VCR vs. PTPN21 + VCR 1.834 ± 0.124 vs. 1.014 ± 0.115, *p* < 0.01; Fig 3E-F). Next, we assessed the phosphorylation of proteins involved in the P53 and JNK signaling pathways in Jurkat cells treated with VCR for 48 h in both the PTPN21 overexpression group and the control group. Phosphorylation of components of the P53 and JNK signaling pathways was enhanced following VCR-induced apoptosis. However, when PTPN21 was overexpressed, the phosphorylation of components of the P53 and JNK signaling pathways was decreased in cells exposed to VCR (Fig 3G-H). In summary, PTPN21 downregulated GADD45A and the phosphorylation of proteins involved in the P53 and JNK signaling pathways.

### The restoration of GADD45A reversed the antiapoptotic effect of PTPN21

**Fig 4:**
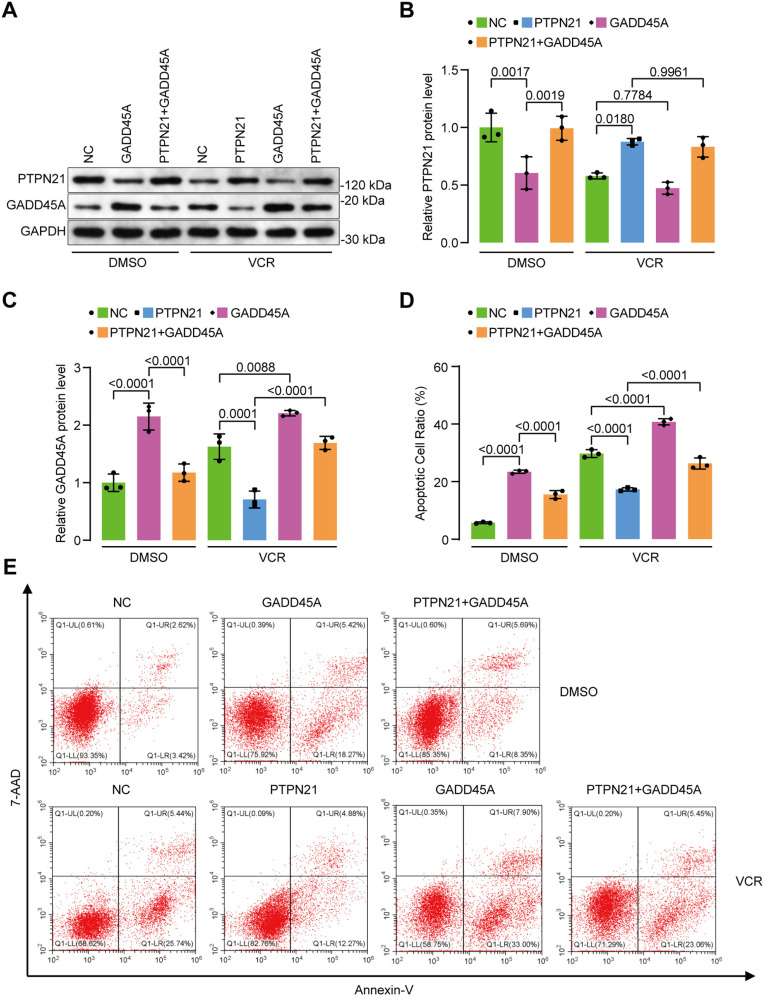
Reversal of the antiapoptotic effect of PTPN21 through the restoration of GADD45A expression. **(A)** Western blot analysis of PTPN21 and GADD45A protein levels. These cells were treated with 5 μM VCR for 48 h or DMSO as a control. **(B-C)** Protein data analysis for **(A)** (n = 3). **(D)** Apoptosis data analysis for **(E)** (n = 3). **(E)** Flow cytometry analysis of apoptosis in these cells. The data are presented as the mean ± standard deviation, and a p value <0.05 indicated statistical significance.

**Fig 5:**
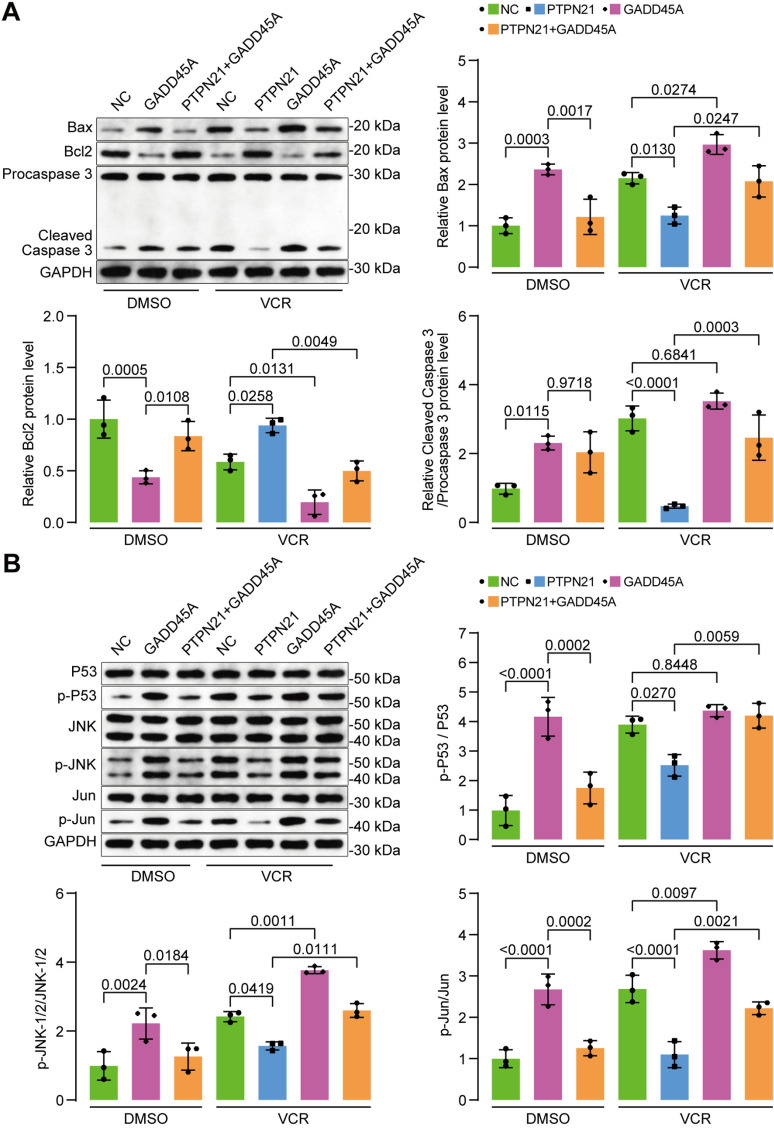
The influence of GADD45A overexpression on apoptosis-associated proteins and the JNK signaling pathway. **(A)** Western blot results showing the expression of apoptosis-associated proteins, including Bax, Bcl-2, and caspase 3 (n = 3). **(B)** Western blot analysis of the phosphorylation status of P53 and proteins in the JNK signaling pathway (n = 3). The data are presented as the mean ± standard deviation, and a p value <0.05 indicated statistical significance.

To confirm the pivotal role of GADD45A in PTPN21-mediated antiapoptotic effects in acute lymphoblastic leukemia (ALL) cells, we restored the expression of GADD45A through lentiviral transfection, while cells transfected with the pHIV7-SFFV-GFP vector served as a control. The overexpression of GADD45A directly downregulated PTPN21 (NC+DMSO vs. PTPN21 + DMSO, 1.025 ± 0.054 vs. 0.694 ± 0.062, *p* < 0.01; Fig 4A-C) and induced apoptosis (NC+DMSO vs. GADD45A+DMSO, 5.720 ± 0.286 vs. 23.440 ± 0.568, NC + VCR vs. GADD45A+VCR, 29.750 ± 1.365 vs. 40.790 ± 1.089, *p* < 0.0001; Fig 4D-E). The overexpression of GADD45A also enhanced the expression of Bax and cleaved caspase 3 but inhibited the antiapoptotic protein Bcl-2 (Fig 5A). After 48 hours of treatment with VCR, PTPN21 was downregulated, while GADD45A was upregulated. In PTPN21-overexpressing Jurkat cells, the restoration of GADD45A significantly increased VCR-induced apoptosis (17.31 ± 0.58 vs. 26.32 ± 1.93, *p* < 0.0001; Fig 4D-E). The restoration of GADD45A also enhanced the expression of Bax and cleaved caspase 3, but inhibited expression of the antiapoptotic protein Bcl-2 (Fig 5A).

Furthermore, the overexpression of GADD45A increased the phosphorylation of proteins involved in the P53 and JNK signaling pathways. In PTPN21-overexpressing Jurkat cells, the restoration of GADD45A increased the phosphorylation of P53 and JNK signaling pathway components in cells treated with VCR (Fig 5B). These results suggest that the restoration of GADD45A reverses the antiapoptotic effect of PTPN21 by regulating the JNK signaling pathway.

### PTPN21 overexpression reversed VCR-induced cell cycle arrest at the G2/M phase

**Fig 6:**
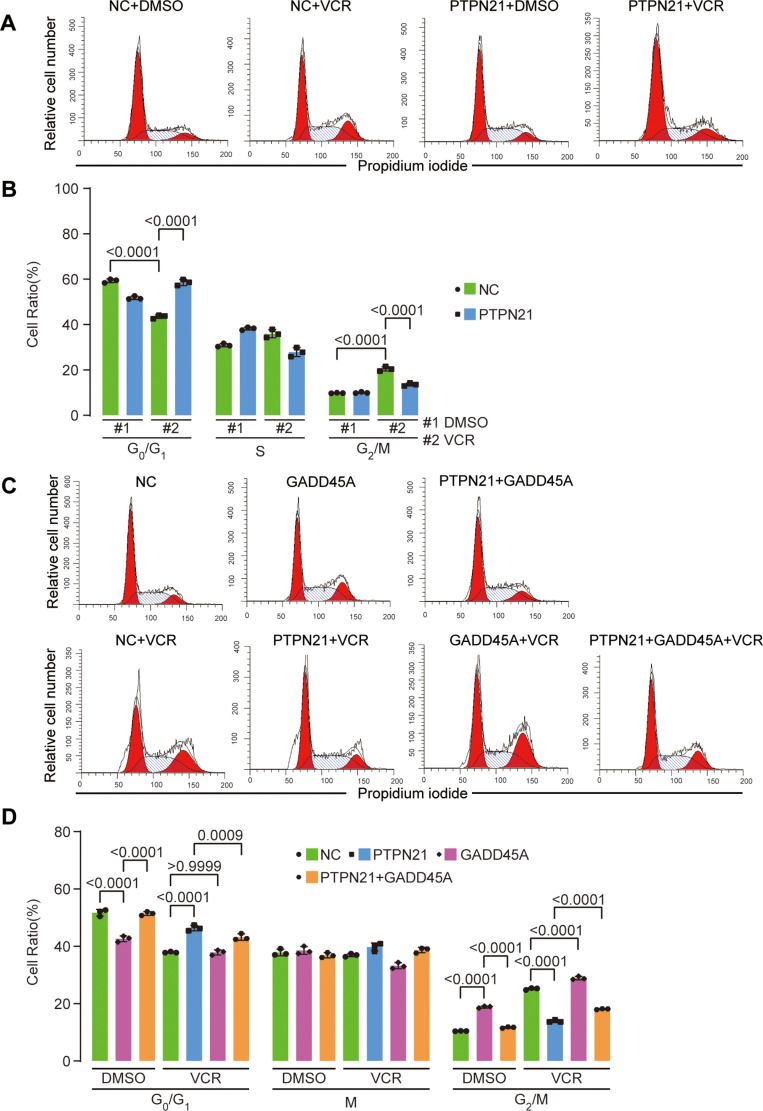
The impact of PTPN21 overexpression on cell cycle progression during VCR treatment. **(A)** Flow cytometry detection of the Jurkat cell cycle after PTPN21 overexpression under VCR conditions. **(C)** Flow cytometry analysis of Jurkat cell cycle after overexpression of PTPN21 and/or GADD45A with/without VCR. (B) and (D) summary of data from (A) and **(C)**, respectively (n = 3). The data are presented as the mean ± standard deviation, and a p value <0.05 indicated statistical significance.

We analyzed the cell cycle distribution of VCR-treated Jurkat cells using FACS, while cells transfected with the pHIV7-SFFV-GFP vector served as a control. VCR strongly induced G2/M cell cycle arrest (G1%: NC+DMSO, 59.28 ± 0.83; NC + VCR, 43.51 ± 0.82; *p* < 0.0001; G2/M%: NC+DMSO, 9.82 ± 0.13; NC + VCR, 20.49 ± 0.98; *p* < 0.0001) (Fig 6A-B). In PTPN21-overexpressing cells treated with VCR, the percentage of cells in G1 phase increased, while that of cells in G2/M phase decreased (G1%: PTPN21 + VCR, 58.56 ± 1.36; NC + VCR, 43.51 ± 0.82; *p* < 0.0001; G2/M%: PTPN21 + VCR, 13.58 ± 0.66; NC + VCR, 20.49 ± 0.98; *p* < 0.0001) (Fig 6A-B). The restoration of GADD45A in PTPN21-overexpressing cells caused G2/M cell cycle arrest (Fig 6C-D). These results indicate that PTPN21 increases the proportion of cells in G1 phase and inhibits G2/M cell cycle arrest to achieve an antiapoptotic effect and that the restoration of GADD45A reinduces G2/M cell cycle arrest to induce apoptosis.

**Fig 7:**
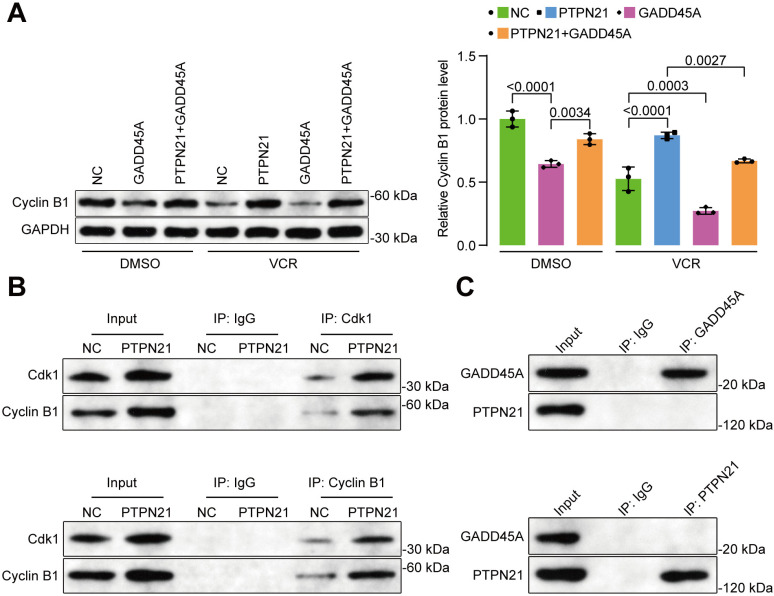
Effects of PTPN21 on the expression of cell cycle proteins and the interaction assay between PTPN21 and GADD45A. **(A)** Western blot results showing Cyclin B1 protein expression (n = 3). **(B)** Co-IP assay was performed to detect the effects of PTPN21 on Cdk1 and Cyclin B1 interactions. **(C)** Co-IP assay was performed to detect the interactions between PTPN21 and GADD45A. The data are presented as the mean ± standard deviation, and a p value <0.05 indicated statistical significance.

In addition, Western blot experiments revealed that VCR significantly reduced the expression of Cyclin B1, which indicates a blockade of the G2/M phase of the cell cycle (Fig 7A). Conversely, Cyclin B1 levels were notably increased following PTPN21 overexpression, but this effect was mitigated by GADD45A, which is consistent with our flow cytometry results that showed cell cycle progression (Fig 7A). Additionally, Co-IP experiments indicated that an increased amount of interaction between Cdk1 and Cyclin B1 could be observed due to elevated Cdk1 expression resulting from PTPN21 overexpression (Fig 7B), which suggests that PTPN21 may accelerate the G2/M phase transition, as corroborated by earlier experiments. However, unexpectedly, the Co-IP experimental results showed that there was no interaction between GADD45A and PTPN21 proteins (Fig 7C).

## Discussion

In this study, we observed that high PTPN21 expression hindered acute lymphoblastic leukemia (ALL) cell apoptosis induced by the chemotherapeutic drugs vincristine (VCR) and daunorubicin (DNR). Our RNA sequencing analysis highlighted the significant roles of GADD45A and the JNK signaling pathway in this process. PTPN21 has been previously linked to the development of various cancers. Our initial insight into the connection between PTPN21 and ALL was derived from the work of Xiao et al., who suggested that PTPN21 mutations might be associated with posttransplantation recurrence [14]. Subsequent in vitro experiments confirmed that the endogenous PTPN21-CDSlong isoform inhibits the susceptibility of ALL cells to NK cell-mediated lysis [15]. Studies have shown that overexpression of PTPN21 can accelerate the proliferation of ALL cells upon EGF stimulation, an effect mediated through the Src/MAPK signaling pathway [32]. This suggests that PTPN21 may play a promotive role in the disease progression of ALL, particularly in terms of cell proliferation. Therefore, inhibitory strategies targeting PTPN21 may emerge as effective means to control the progression of ALL. Although the expression of PTPN21 has been correlated with immune infiltration levels in various cancers, the specific mechanisms of its action in ALL remain to be thoroughly explored. Notably, the correlation between PTPN21 and multiple immune cell markers, such as CD4 + T cells and macrophages, hints at its potential role in regulating the tumor immune microenvironment, paving the way for the development of novel therapeutic strategies [33]. Currently, treatment strategies for ALL encompass tyrosine kinase inhibitors (TKIs) and CAR T-cell therapy, among others [34]. As a potential therapeutic target, PTPN21 holds promise for combination therapy with these existing treatments to enhance therapeutic efficacy. For instance, inhibiting PTPN21 to curb the proliferation of ALL cells, in conjunction with TKIs or other immunotherapies, could further bolster the eradication capabilities against tumor cells. However, it is unknown whether PTPN21 influences the effects of VCR and DNR on ALL. Our findings reveal the pivotal role of PTPN21 in the treatment of ALL with VCR and make a significant contribution to this field. Recent reports have revealed that PTPN21 plays a crucial role in maintaining the homeostasis and biomechanics of hematopoietic stem cells (HSCs) [35]. PTPN21 regulates the phosphorylation of Septin1, which, in turn, sustains cellular mechanics. This function is closely associated with the role of PTPN21 in HSC niche retention and in the preservation of hematopoietic regeneration capacity. Interestingly, these reports demonstrate that PTPN21 deletion in HSCs leads to decreased quiescence and increased apoptosis. However, our results indicate that direct PTPN21 overexpression did not impact apoptosis. This discrepancy may be attributed to the distinct cytological characteristics of stem cells compared with those of regular cell lines. Future investigations should explore the unique role of PTPN21 in stem cell-like cells, particularly through the study of leukemia stem cells.

The RNA sequencing results revealed that GADD45A (growth arrest and DNA damage-inducible α) was downregulated in PTPN21-overexpressing cells after exposure to VCR. GADD45A is a well-known regulator of growth arrest and apoptosis [36]. In our experiments, VCR-induced apoptosis in ALL cells was associated with an increase in GADD45A expression. However, this upregulation of GADD45A was counteracted by PTPN21 overexpression. Subsequent investigations into downstream pathways revealed inhibition of both the JNK pathway and P53 phosphorylation. Conversely, the restoration of GADD45A expression enhanced apoptosis and the phosphorylation of the JNK pathway components and P53. Numerous studies have confirmed the involvement of GADD45A in regulating drug-induced apoptotic processes [37–39], including responses to VCR [40]. Consistent with our findings, a previous study reported that Brca1-induced GADD45A transactivation activates JNK-mediated apoptosis in vitro [41]. This activation occurs through a conformational change induced by the binding of GADD45 to MEKK4 (MTK/MEK kinase 4), which releases the MEKK4 kinase domain from its interaction with an N-terminal autoinhibitory domain. This allows for the recognition and phosphorylation of MKK6 and SEK1, which directly activates the JNK pathway [42]. The JNK pathway has been shown to be involved in various aspects of both the intrinsic and extrinsic pathways of apoptosis [43,44]. Our results align with previous studies that have demonstrated reduced apoptosis when JNK pathway phosphorylation is downregulated. Moreover, alterations in P53 phosphorylation were also observed in our study. Although GADD45A is one of the numerous downstream targets of P53 [45], it has been shown to function in regulating P53. Stress-induced activation of MTK1 leads to rapid phosphorylation and activation of MAPK, which contributes to the activation of several transcription factors, including P53. GADD45A is, in turn, activated by both P53-dependent and P53-independent mechanisms. Ultimately, the newly synthesized GADD45A protein is crucial for maintaining MAPK and P53 activity through a positive feedback loop with MTK1 [46]. This finding suggests that P53 may also participate in PTPN21-mediated inhibition of VCR-induced apoptosis by interacting with the JNK pathway.

For decades, it has been well established that VCR induces apoptosis by arresting cells in G2/M phase [47]. Furthermore, it has been confirmed that GADD45A can interact with Cdk1, which potentially prevents its binding to Cyclin B1, thus inhibiting Cdk1 activity and causing cell cycle arrest at the G2/M checkpoint [48]. Consequently, we hypothesize that PTPN21 inhibits G2/M cell cycle arrest by downregulating GADD45A, which consequently drives cells back into G1 phase, and in turn, inhibits VCR-induced apoptosis. Intriguingly, in the GADD45A overexpression experiment, we observed the inhibition of PTPN21, suggesting a possible negative feedback loop between PTPN21 and GADD45A. However, surprisingly, our research found that there was no interaction between PTPN21 and GADD45A proteins, indicating that PTPN21 may indirectly affect the processing, stability, or translation of GADD45A mRNA by influencing other molecules.

Although this study demonstrated that PTPN21 could regulate the expression of GADD45A and consequently influence VCR-induced apoptosis in ALL cells, several limitations were identified. Firstly, despite our study unveiling a regulatory relationship between PTPN21 and GADD45A, the absence of a direct protein-protein interaction between them suggests that PTPN21 may regulate the processing, stability, or translation of GADD45A mRNA indirectly through the influence of other molecules. Therefore, further exploration is required to elucidate the specific molecular pathway through which PTPN21 modulates GADD45A expression. Secondly, our findings indicate that the expression of PTPN21 may impact the therapeutic efficacy of vincristine (VCR) in acute lymphoblastic leukemia (ALL) cells, potentially contributing to the development of drug resistance. However, this study did not comprehensively evaluate PTPN21 as a potential therapeutic target in VCR-resistant ALL cell lines. Consequently, future research should prioritize the development and utilization of VCR-resistant ALL cell lines to further investigate the role of PTPN21 in drug resistance and assess its potential as a therapeutic target. Lastly, it is important to note that all experiments conducted in this study were performed in vitro, which may not fully capture the complexity of the in vivo environment. Therefore, differences may arise between in vivo and in vitro experimental results. Subsequent studies will validate and investigate these findings through in vivo experiments.

In conclusion, our study revealed that the overexpression of PTPN21 in acute lymphoblastic leukemia (ALL) cells markedly impairs the induction of apoptosis by DNR and VCR. Our in-depth analysis further reveals that the primary mechanism underlying this effect involves the suppression of the growth arrest and DNA GADD45A, coupled with the inhibition of the JNK signaling pathway. These findings not only emphasize the crucial significance of PTPN21 in the pathophysiology of ALL but also accentuate its potential as an exceptionally promising therapeutic target for the treatment of this condition.

## Supporting information

S1 Fig The schematic diagram of the pHIV7/SFFV-GFP plasmid. (TIF)

S2 Fig Flow cytometry control. (A) 7-AAD single-color control. (B) APC single-color control. (C) Negative control.
(TIF)

S1 File Supplementary material. The protocol of construction of eukaryotic expression vector plasmid for PTPN21 gene and flow cytometry experiments.
(DOCX)

S2 File Uncropped images for all blot results. (PDF)
