# Supplementary figures and images for: PTPN21 inhibits cell apoptosis of acute lymphoblastic leukemia induced by chemotherapeutic agents via GADD45A and JNK signaling pathway
Source: PLoS One. 2025 Apr 30;20(4):e0322273. doi: 10.1371/journal.pone.0322273 (PMC12043166; doi:10.1371/journal.pone.0322273)

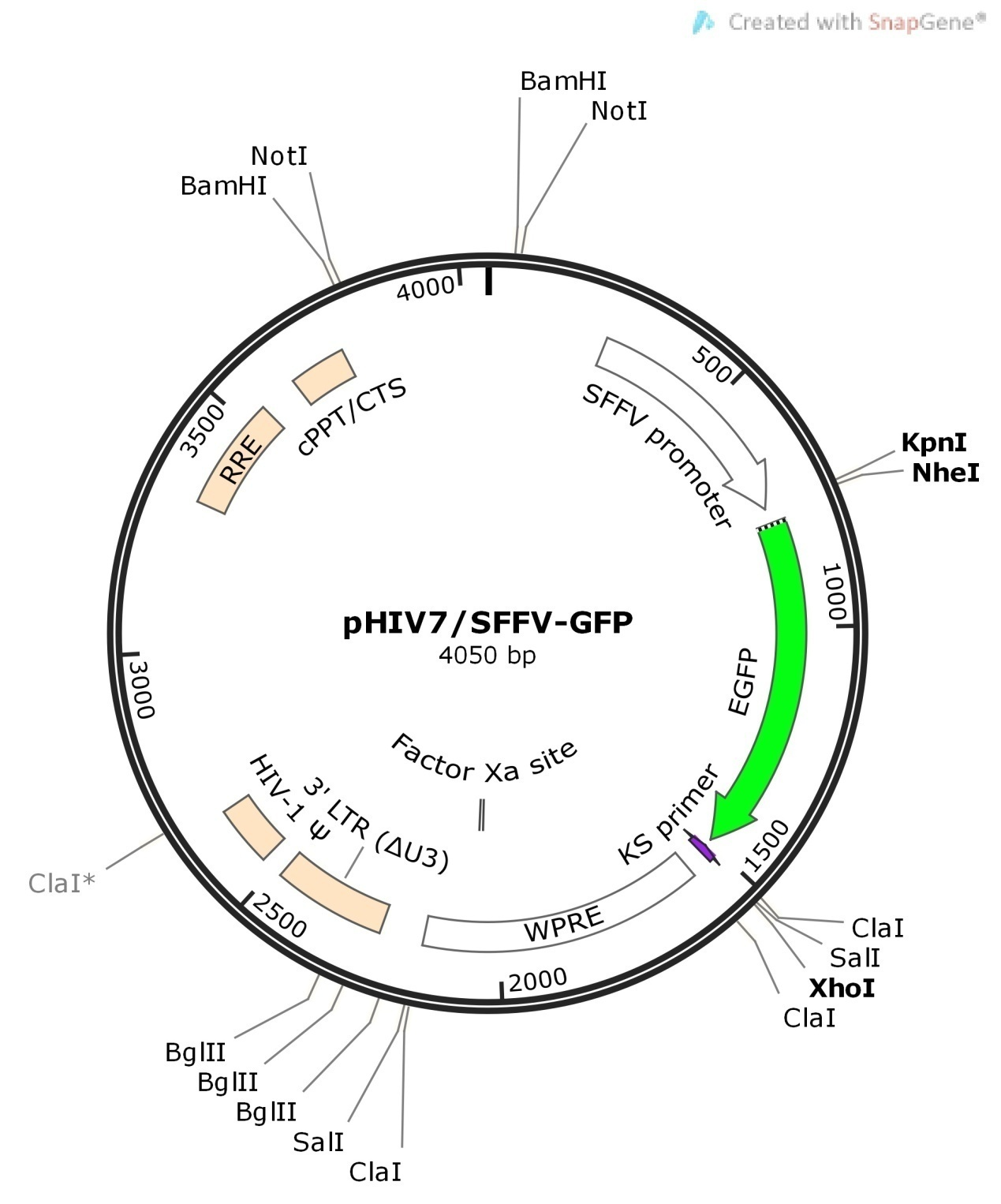

Supplement: S1 Fig — (TIF) [file pone.0322273.s001.tif]

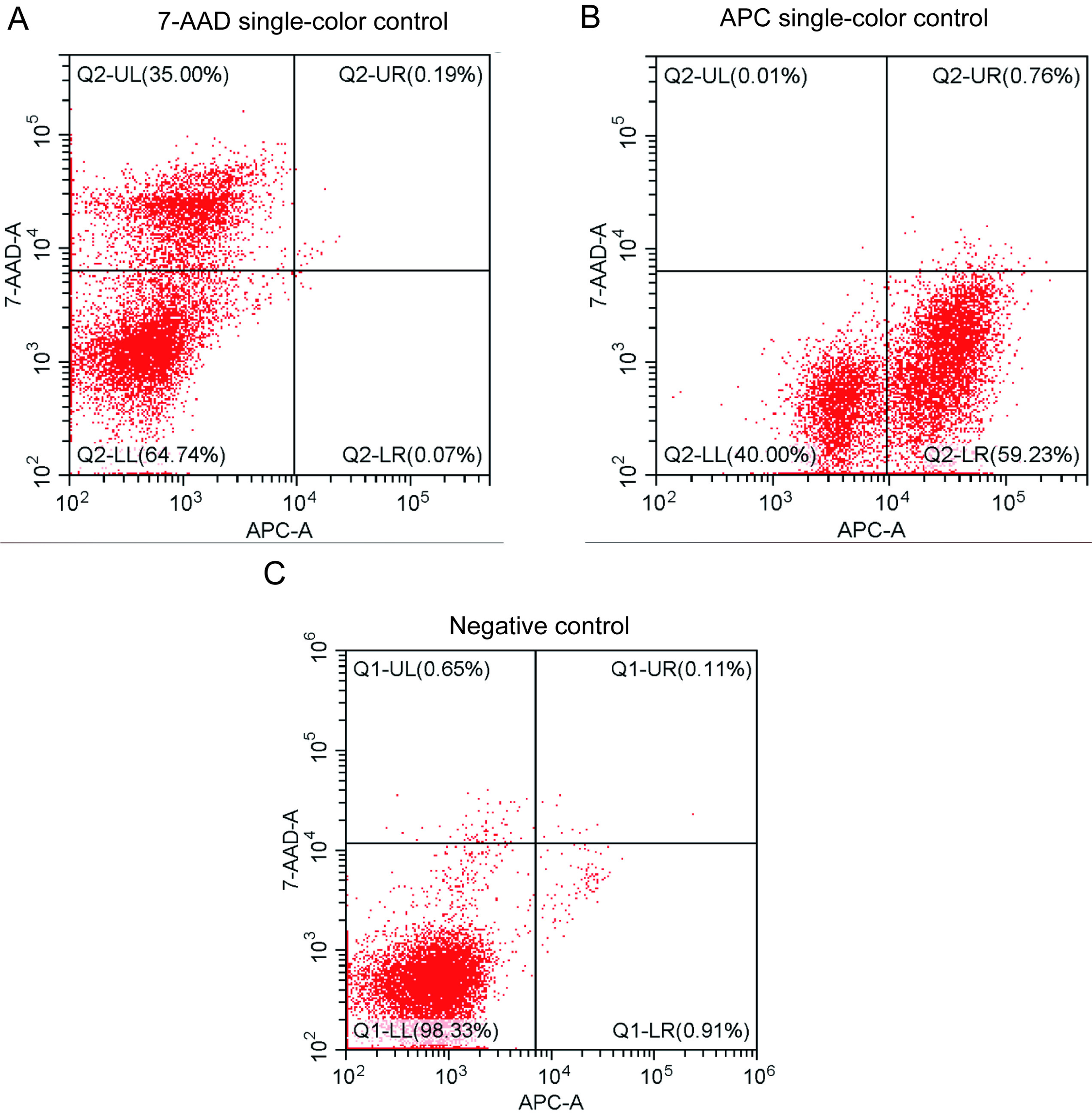

Supplement: S2 Fig — (A) 7-AAD single-color control. (B) APC single-color control. (C) Negative control. (TIF) [file pone.0322273.s002.tif]
