# Supplementary material for: PTPN21 inhibits cell apoptosis of acute lymphoblastic leukemia induced by chemotherapeutic agents via GADD45A and JNK signaling pathway
Source: PLoS One. 2025 Apr 30;20(4):e0322273. doi: 10.1371/journal.pone.0322273 (PMC12043166; doi:10.1371/journal.pone.0322273)
Supplement: S1 File — The protocol of construction of eukaryotic expression vector plasmid for PTPN21 gene and flow cytometry experiments. (DOCX) [file pone.0322273.s003.docx]

**Construction of Eukaryotic Expression Vector Plasmid for PTPN21 Gene**

(1) TA Cloning

The target sequence, 3×FLAG Tag, was analyzed for complex secondary structures and repetitive sequences. Based on the analysis, single-stranded oligos were designed and synthesized. PCR was utilized to assemble the oligos into a complete gene sequence of 3×FLAG Tag, including a stop codon, totaling 69 bp. The assembled sequence was cloned into the pMD18-T vector and transformed into DH5α competent cells. Sequencing was performed to confirm the accuracy of the inserted sequence.

(2) Construction of Eukaryotic Expression Vector Plasmid

The SFFV promoter, amplified from the pHIV7/SFFV-GFP plasmid, was fused with the PCR-amplified PTPN21 gene sequence in the order of SFFV-PTPN21. This fused fragment was then joined with the previously synthesized 3×FLAG Tag in the order of SFFV-PTPN21-3×FLAG. The pHIV7/SFFV-GFP plasmid was digested with BamHI, and the larger fragment was recovered for subsequent vector construction. The fused SFFV-PTPN21-3×FLAG fragment was digested with BglII and ligated into the BamHI-digested pHIV7/SFFV-GFP large fragment to generate the pHIV7/SFFV-PTPN21-3×FLAG plasmid. The specific methods are as follows:

a. Three pairs of primers were designed to amplify and fuse the corresponding fragments. The primers included:SFFV-P-BglII-F, SFFV-P-R, PTPN21-F, PTPN21-R, FLAG-F, and FLAG-BglII-R. The amplified and fused fragments were recovered for further experiments.

b. The fused fragments were digested with BglII at 37°C for 2 hours in a reaction mixture containing 5 μl of 10× buffer, 10 μl of fused fragments (approximately 1 μg), 1 μl of BglII enzyme, and 34 μl of ddH2O.

c. The pHIV7/SFFV-GFP plasmid was digested with BamHI at 37°C for 2 hours in a reaction mixture containing 5 μl of 10× buffer, 2 μl of plasmid (approximately 1 μg), 1 μl of BamHI enzyme, and 42 μl of ddH2O.

d. The digested fragments were recovered by electrophoresis and ligated with the vector using T4 DNA ligase at room temperature for 2 hours. The ligation reaction mixture contained 1 μl of ligation buffer, 1 μl of vector, 2 μl of ligated fragments, 0.5 μl of T4 DNA ligase (400 U/μl), and 5.5 μl of ddH2O.

e. Five microliters of the ligation product were transformed into DH5α competent cells. Multiple clones were picked from the transformation plate and sequenced to confirm the identity of the expression vector.

**Flow cytometry experiment method**

Negative control: Normal cells without the addition of Annexin V and 7-AAD.

APC single-color control: 1 μL of VCR (5 μM) combined with 5 μL of Annexin V single staining.

7-AAD single-color control: 1 μL of VCR (5 μM) combined with 5 μL of 7-AAD single staining.

Gating Strategies: The gain settings were adjusted to 11 for FSC (Forward Scatter) and 1 for SSC (Side Scatter). The threshold was set automatically, with the width channel configured for FSC.
