# Supplementary material for: PTPN21 inhibits cell apoptosis of acute lymphoblastic leukemia induced by chemotherapeutic agents via GADD45A and JNK signaling pathway
Source: PLoS One. 2025 Apr 30;20(4):e0322273. doi: 10.1371/journal.pone.0322273 (PMC12043166; doi:10.1371/journal.pone.0322273)

Uncropped gels for Western Blots in Figure 1A

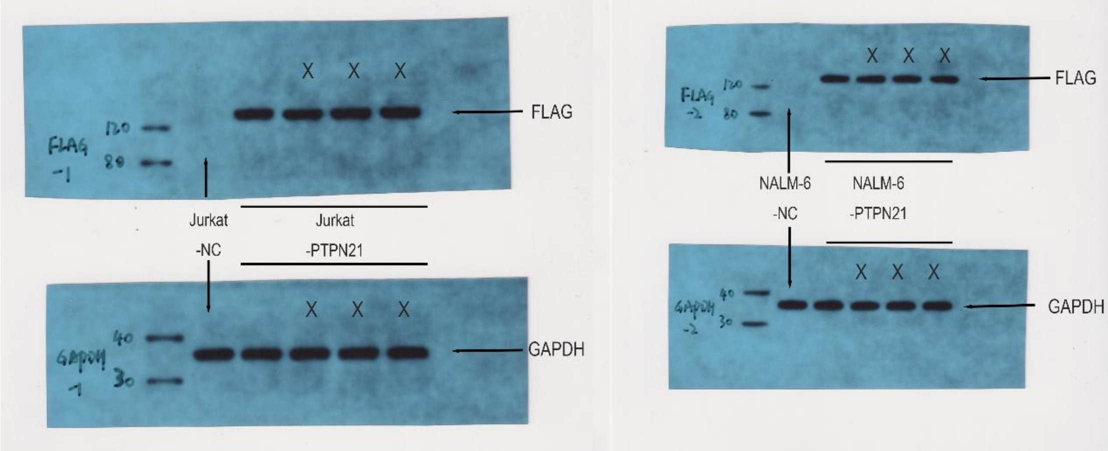

Uncropped gels for Western Blots in Figure 2C

1=NC+DMSO

2=PTPN21+DMSO

3=NC+VCR

4=PTPN21+VCR

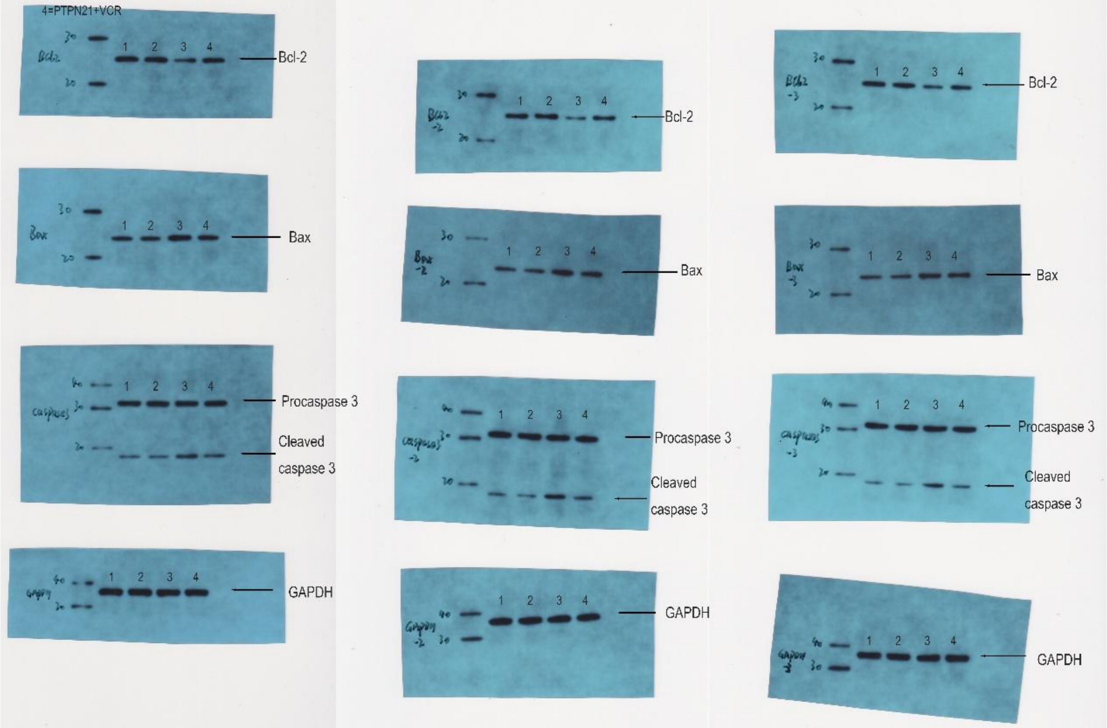

Uncropped gels for Western Blots in Figure 3E

1=NC+DMSO

2=NC+VCR

3=PTPN21+DMSO

4=PTPN21+VCR

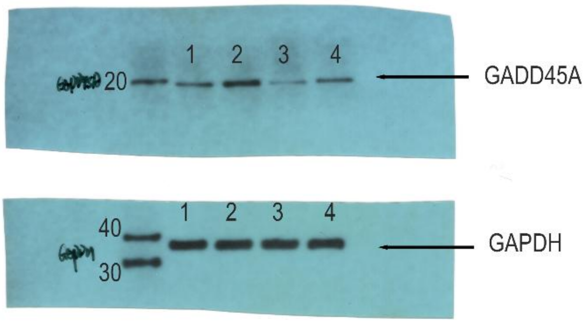

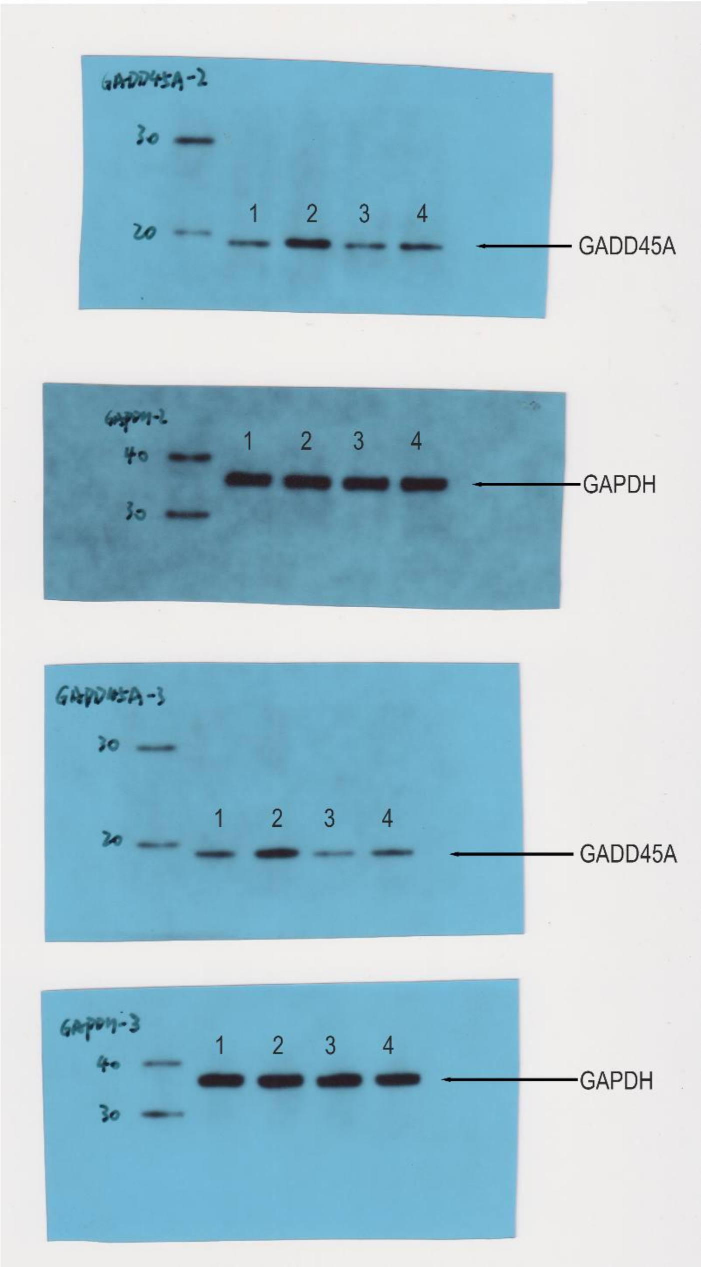

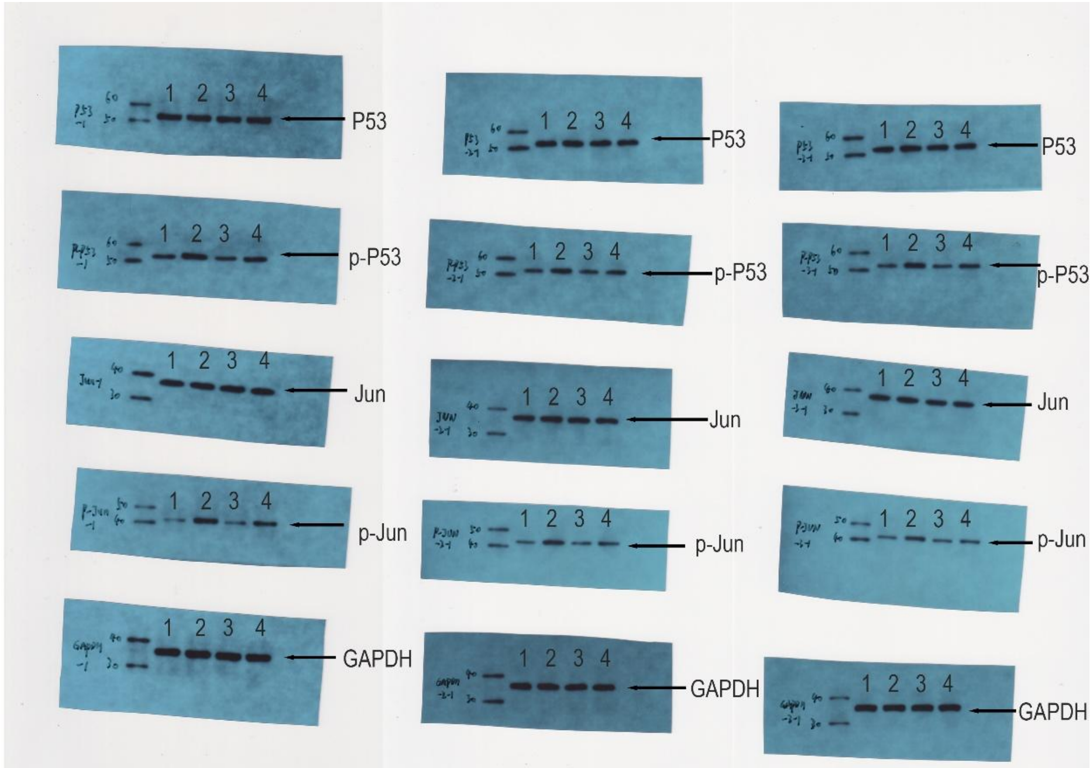

Uncropped gels for Western Blots in Figure 3G

1=NC+DMSO

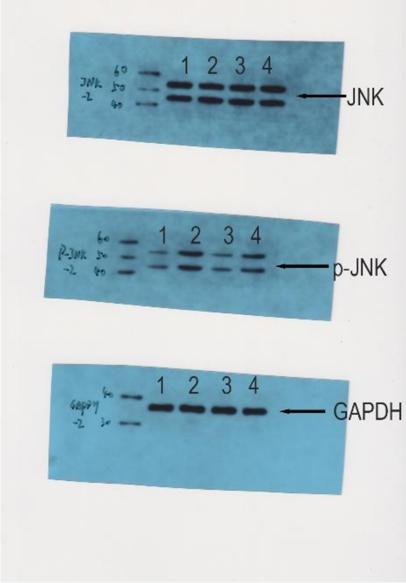

2=NC+VCR

3=PTPN21+DMSO

4=PTPN21+VCR

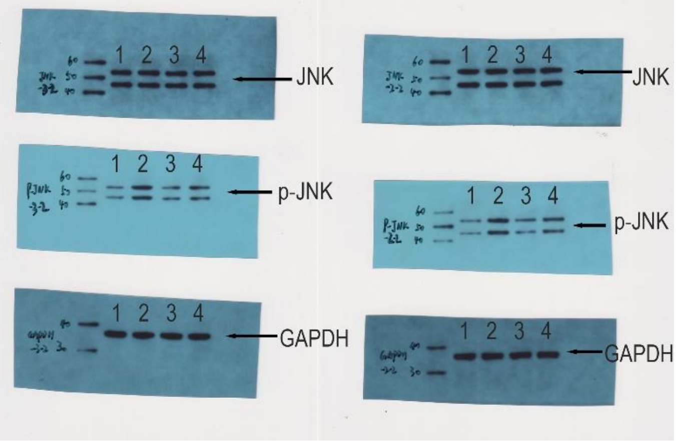

Uncropped gels for Western Blots in Figure 4A

1=NC+DMSO

2=GADD45A+DMSO

3=PTPN21+GADD45A+DMSO

4=NC+VCR

5=PTPN21+VCR

6=GADD45A+VCR

7=PTPN21+GADD45A+VCR

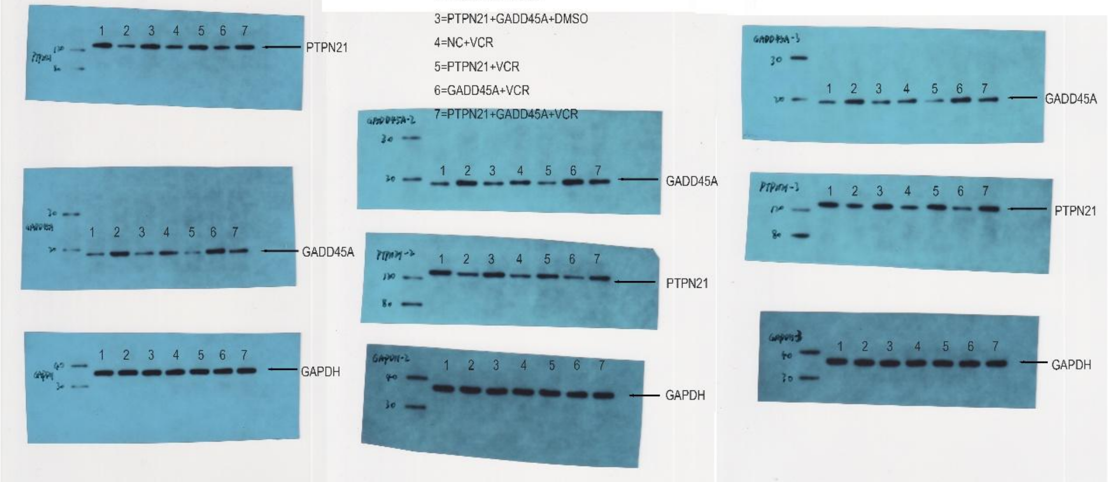

Uncropped gels for Western Blots in Figure 5A

1=NC+DMSO

2=GADD45A+DMSO

3=PTPN21+GADD45A+DMSO

4=NC+VCR

5=PTPN21+VCR

6=GADD45A+VCR

7=PTPN21+GADD45A+VCR

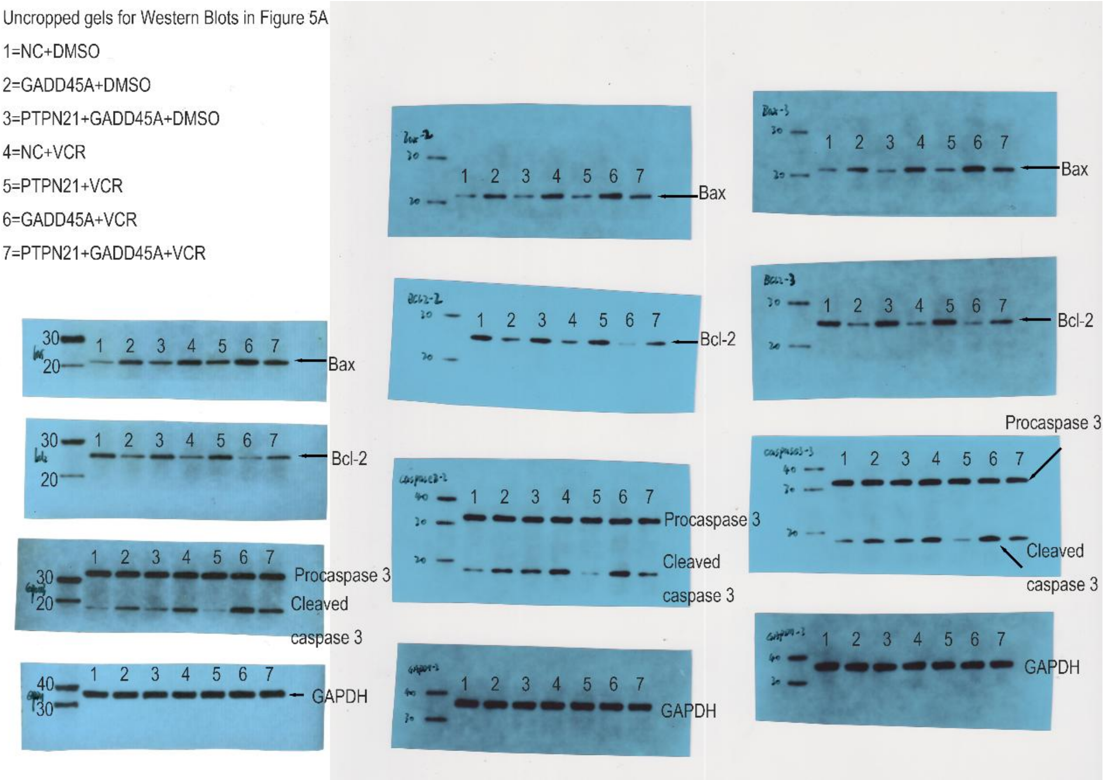

Uncropped gels for Western Blots in Figure 5B

1=NC+DMSO

2=GADD45A+DMSO

3=PTPN21+GADD45A+DMSO

4=NC+VCR

5=PTPN21+VCR

6=GADD45A+VCR

7=PTPN21+GADD45A+VCR

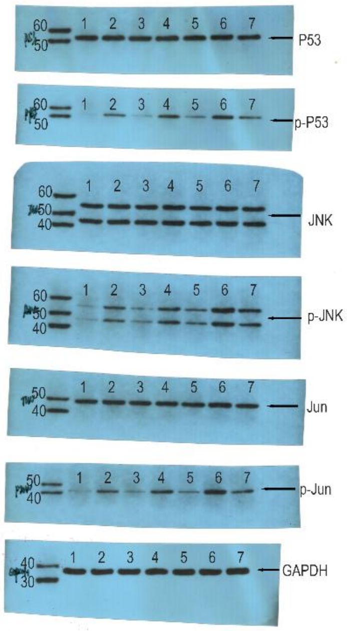

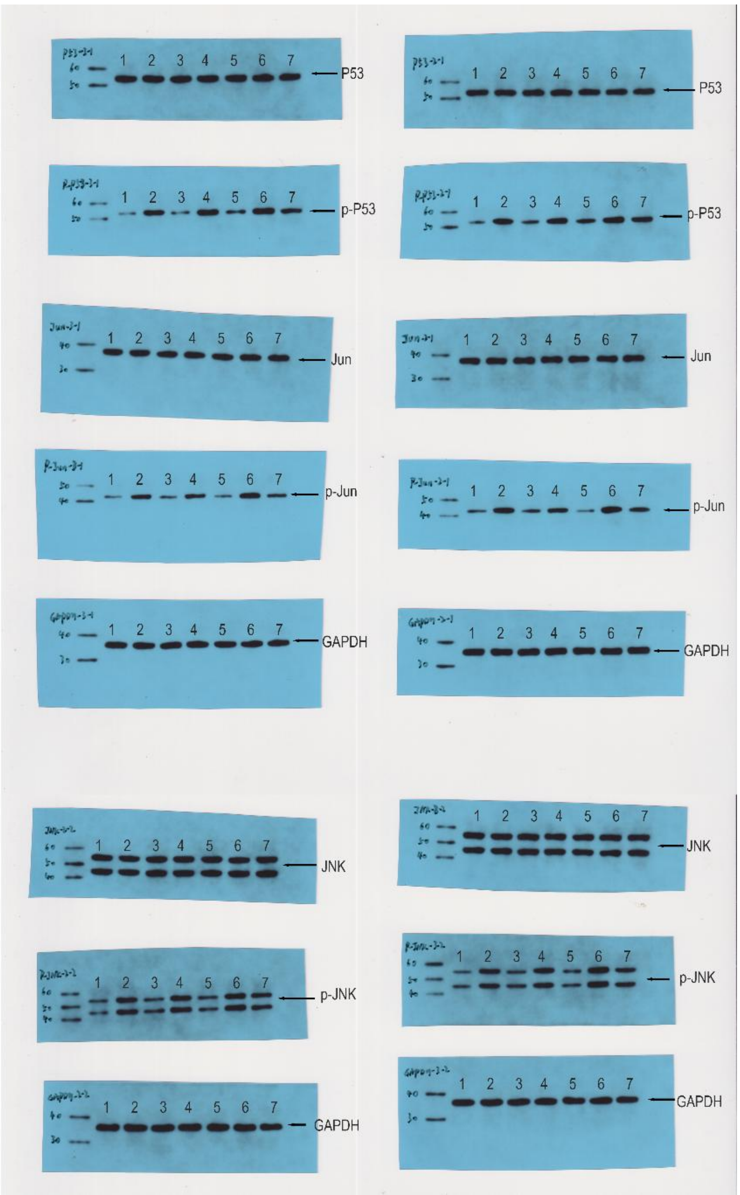

Uncropped gels for Western Blots in Figure 7A

1=NC+DMSO

2=GADD45A+DMSO

3=PTPN21+GADD45A+DMSO

4=NC+VCR

5=PTPN21+VCR

6=GADD45A+VCR

7=PTPN21+GADD45A+VCR

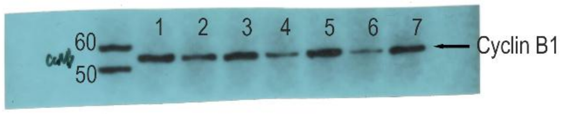

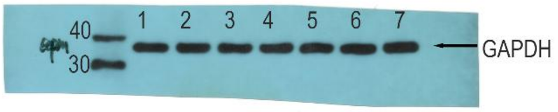

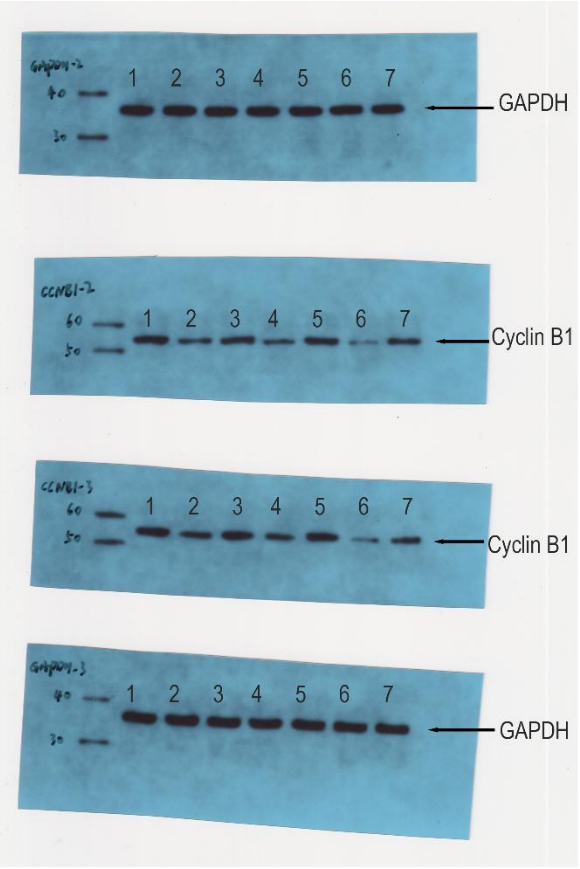

Uncropped gels for Co-IP in Figure 7B

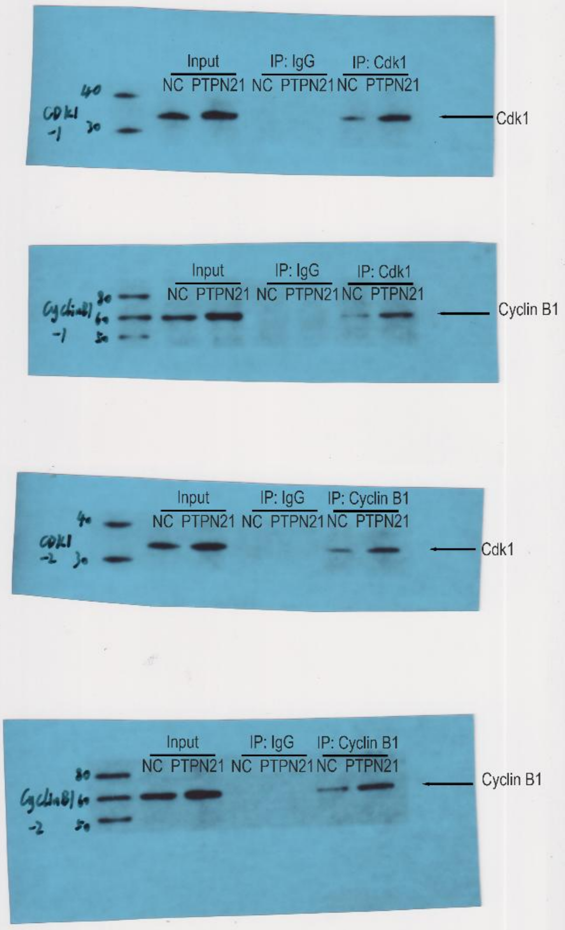

Uncropped gels for Co-IP in Figure 7C

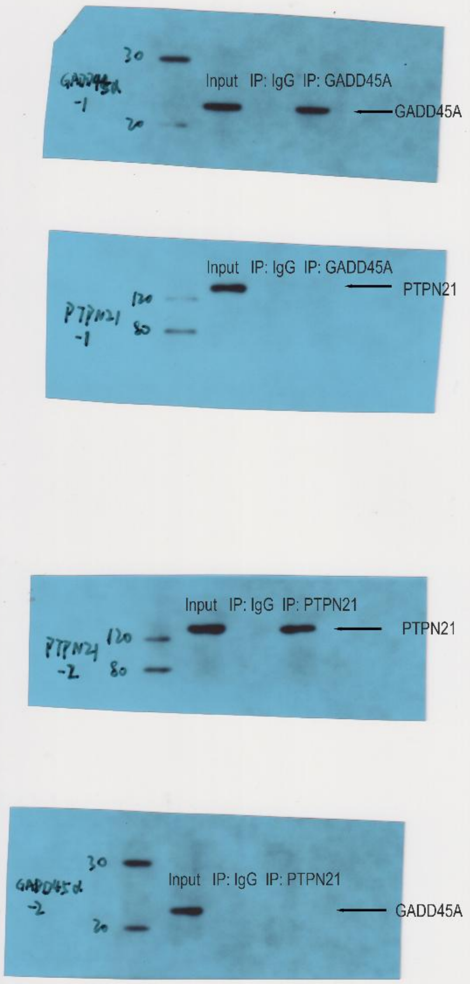

Supplement: S2 File — (PDF) [file pone.0322273.s004.pdf]
